# Sarcopenia and Variation in the Human Leukocyte Antigen Complex

**DOI:** 10.1093/gerona/glz042

**Published:** 2019-02-17

**Authors:** Garan Jones, Luke C Pilling, Chia-Ling Kuo, George Kuchel, Luigi Ferrucci, David Melzer

**Affiliations:** 1 Epidemiology and Public Health Group, University of Exeter Medical School; 2 Biostatistics Center, CT Institute for Clinical &Translational Science, Department of Community Medicine and Health Care, University of Connecticut Health Center, Farmington; 3 Center on Aging, University of Connecticut Health Center, Farmington; 4 National Institute on Aging, Baltimore, Maryland

**Keywords:** UK Biobank, Autoimmune, Inflammation, Muscle

## Abstract

**Background:**

Aging is characterized by chronic inflammation plus loss of muscle mass and strength, termed sarcopenia. Human leukocyte antigen (HLA) types are drivers of autoimmune disease, although with limited penetrance. We tested whether autoimmune diagnoses are associated with sarcopenia, and whether HLA types and related genetic variants are associated with sarcopenia in autoimmune disease-free older people.

**Methods:**

Data were collected from 181,301 UK Biobank European descent volunteers aged 60–70 with measured hand grip strength and impedance. Logistic regression analysis estimated HLA type and sarcopenia associations, adjusted for confounders and multiple testing.

**Results:**

Having any autoimmune diagnosis was associated with sarcopenia (odds ratio [OR] 1.83, 95% confidence interval (CI) 1.74–1.92, *p* = 4.0*10^−125^). After excluding autoimmune diagnoses, 6 of 100 HLA types (allele frequency >1%) were associated with sarcopenia (low grip strength and muscle mass). Having two HLA-DQA1*03:01 alleles increased odds of sarcopenia by 19.3% (OR 1.19, CI 1.09–1.29, *p* = 2.84*10^–5^), compared to no alleles. Having ≥6 of the 12 HLA alleles increased sarcopenia odds by 23% (OR 1.23, CI 1.12–1.35, *p* = 7.28*10^–6^). Of 658 HLA region non-coding genetic variants previously implicated in disease, 4 were associated with sarcopenia, including rs41268896 and rs29268645 (OR 1.08, CI 1.05–1.11, *p* = 1.06*10^–8^ and 1.07, CI 1.04–1.09, *p* = 1.5*10^–6^, respectively). Some HLA associations with sarcopenia were greater in female participants.

**Conclusion:**

Autoimmune diagnoses are strongly associated with sarcopenia in 60- to 70-year olds. Variation in specific HLA types and non-coding single nucleotide polymorphisms is also associated with sarcopenia in older carriers free of diagnosed autoimmune diseases. Patients with sarcopenia might benefit from targeted treatment of autoimmune processes.

Immunosenescence and “Inflammaging” are characterized by a low-grade chronic pro-inflammatory state associated with aging, which results from an imbalance between the inflammatory and anti-inflammatory networks (1,2). The human leukocyte antigens (HLAs) have a major role in mediating the chronic inflammatory pathway in autoimmune disease via antigen presentation to the CD4^+^/8^+^ T cells (3). The HLA complex has long been associated with autoimmune disorders and infectious diseases (4). Examples of associations between certain HLA types and autoimmune disease include HLA-B*27 and spondyloarthritis (SpA), HLA-B*51 and Behcet’s disease (5), and HLA-DRB1*15:01 and multiple sclerosis (6,7). Many autoimmune diseases directly impact on muscle strength and physical functioning; however, autoimmune diseases are generally not monogenic or fully penetrant, but rather are complex polygenic diseases influenced by environmental factors (despite having more than 110 linked genes, excluding HLA types, the heritability of multiple sclerosis is approximately 28% (8)). We hypothesize, therefore, that both those with diagnosed autoimmune conditions and carriers of certain HLA types without diagnosed autoimmune diseases may have increased risk of age-related conditions.

Sarcopenia, loss of muscle mass and strength during aging, is associated with reduced physical functioning and increased risks of morbidity (including falls and fractures) and mortality (9,10). The European Working Group on Sarcopenia in Older People (EWGSOP) defines age-related sarcopenia as a disease associated with both low muscle mass and low muscle function (9). The Baltimore Longitudinal Study of Aging (BLSA) found evidence that this can occur as early as age 40 in both men and women (11), with progressive decline over time. However, studies of age-related sarcopenia commonly use an age range of 60 or older, to exclude causes of muscle weakness earlier in life (12). Previous studies have presented evidence for the role of inflammation in sarcopenia (13,14), and it is becoming apparent that contributors to disease vary in penetrance from Mendelian disorders to complex diseases (15). We hypothesize that certain HLA types may increase risks for sarcopenia, possibly via undiagnosed or subclinical autoimmune processes. While muscle loss is a classical feature of multiple sclerosis and some other autoimmune conditions, little is known of whether carrying certain HLA types predisposes to autoimmunity which in turn accelerates muscle loss in later life.

We aimed to test associations between diagnosed autoimmune conditions and sarcopenia in the exceptionally large UK Biobank study. We also aimed to test associations between common HLA types and sarcopenia in individuals independently of any diagnosed autoimmune condition, using three definitions of sarcopenia (EWGSOP definition published in 2010—low grip strength and lean muscle mass combined, low grip strength only, and lean muscle mass only). We hypothesize that the presence of certain HLA types are more common in sarcopenic individuals in later life, even in the absence of diagnosed autoimmune disease.

## Methods

We used data from 451,447 UK Biobank participants of European descent, confirmed by principal components analyses of genome-wide genetic information from a selection of participants who self-identified as “white European” after filtering for age (60–70) and any diagnosis of autoimmune disease, 181,301 participants were included in the final analysis (16). The UK Biobank is a volunteer study where participants visited one of 22 assessment centers across the United Kingdom. A range of physiological and questionnaire data was collected, including genetic data from blood draws (17). Genotyping data were generated on the initial ~50,000 participants using the Affymetrix UK BiLEVE Axiom array and the ~450,000 participants of the remaining cohort were genotyped using the Affymetrix UK Biobank Axiom array—the two arrays sharing over 95% similarity (http://www.ukbiobank.ac.uk).

### Phenotype Definitions

Sarcopenia was classified using the EWGSOP definition (9), based on having both low hand grip strength and skeletal muscle mass index (SMI). For grip strength, the highest value from left hand grip strength and right hand grip strength was taken as the selected metric. Low grip strength was defined as under 30 kg in males and under 20 kg in females, as measured by Jamar J00105 hydraulic hand dynamometer.

Skeletal muscle mass (SMM) was calculated using the following equation from Janssen and coworkers (18):

$$\begin{array}{cc} \mathrm{SMM}\;(\mathrm{kg})=\; & [(Ht^{2}|R\times 0.401)+\;(gender\times 3.825)\\ & +(age\times -0.071)]+5.102 \end{array}$$

where Ht is standing height in centimeters measured at the initial assessment; *R* is Bioelectrical Impedance Analysis (BIA) resistance in ohms for the whole body taken by Tanita BC418MA body composition analyzer at the initial assessment visit; for sex, men = 1 and women = 0; and age is in years. SMI was then calculated from the SMM.

$$\begin{array}{c} SMI\;=\;SMM/Ht^{2}\;(in\;meters^{\;}) \end{array}$$

Low SMI was defined by Janssen and coworkers as under 8.87 in males and under 6.42 in females. Analysis was restricted to the age range of 60–70. We excluded a small number of participants with maximum grip strength or lean mass (from BIA) greater than 4 *SD*s from the mean (SMI > 46.61; maximum grip > 73.91).

Three definitions of sarcopenia were developed based on the EWGSOP classification: both low grip strength and lean muscle mass (grip strength 30 kg for males and 20 kg for females, and low SMI 8.87 for males and 6.42 for females); low grip strength only (grip strength 20 kg for females and 30 kg for males); and low muscle mass only (low SMI 6.42 for females and 8.87 for males). While dual-energy X-ray absorptiometry (DXA) is the preferred alternative to computed tomography and magnetic resonance imaging for estimating muscle mass, EWGSOP deemed that BIA may be considered as a portable alternative to DXA.

The Foundation for the National Institutes of Health (FNIH) definition of sarcopenia (19) based on low grip strength and appendicular lean mass could not be analyzed with the cohort due to less than 0.2%/0.1% of the female/male participants meeting the criteria.

### Autoimmune Disease Exclusions

Some autoimmune diseases are known to influence muscle loss. We aimed to determine whether HLA types were associated with sarcopenia in the absence of diagnosed autoimmune disease, and we therefore excluded participants with a diagnosis at the baseline assessment (self-reported or hospital diagnosed).

A list of 135 (including subgroups) ICD-10 codes for autoimmune diseases were generated from the previous review articles (20) and the codes were used to exclude participants from the initial analysis (Supplementary Table 1). In addition, UK Biobank self-reported data were also used based on the autoimmune conditions in the ICD-10 list. Following the initial analysis, an additional 55 autoimmune diseases associated with the HLA types were added to the exclusion criteria and formed the basis of the secondary analysis (Supplementary Table 2).

Logistic regression was performed on a selected subset of more common autoimmune conditions such as rheumatoid arthritis, multiple sclerosis, coeliac disease, type 1 diabetes, psoriasis, and ulcerative colitis. In order to include as many participants as possible with each condition, all ICD-10 codes belonging to the superclass of each condition were included (e.g., all ICD-10 codes for M06* are included for rheumatoid arthritis), as well as any reported in the UK Biobank data-field, 20002: Non-cancer illness code, self-reported (Supplementary Table 10 for details).

### HLA Imputation

Imputation of HLA types was performed centrally by the UK Biobank team. In brief, HLA*IMP:02 (21) was used to impute the four-digit HLA types from genotype information, with a number of modifications: localization feature turned off; graph sampling error (mS) and graph building error (mB) probabilities were both set to 0.001; and the number of sampled haplotype pairs was set to five (22). Individuals are, therefore, coded as 0, 1, or 2 depending on the number of HLA alleles carried for each gene. The methods allow for imprecise coding (e.g., 1.92) to indicate the confidence in HLA type imputation. Some HLA type codes indicate unknown or other HLA types (e.g., *99:01), and these were not included in analyses. HLA types below 1% frequency were excluded from analyses to account for lower-quality imputation for rarer alleles.

### Statistical Analysis

Logistic regression analyses were performed for each HLA type against sarcopenia, adjusted for age, sex, genotype array, and the first five principal components for ancestry. Cohorts were restricted to the age range of 60–70 and of European descent. Participants with known autoimmune disease were excluded as previously described, based on Hospital Episode Statistics data (ICD-10 codes) and self-reported fields.

HLA types were modeled first assuming an additive effect, and secondly comparing participants with two alleles to those with no alleles. Participants with imprecise HLA imputations were recorded for the second, categorical analysis (i.e., estimated allele dose between 0 and 0.25 set to 0, values between 0.75 and 1.25 set to 1, and finally between 1.75 and 2 to 2; other doses were set as missing due to imprecise imputation). Correction for multiple testing was applied using the Benjamini–Hochberg method, for the additive and recessive models separately as these were independent hypotheses. Statistical analyses were performed in STATA (v14.1) and R (v3.3.2). Charts and figures were generated with package metafor (v2.0).

After analyzing each HLA type for its association with sarcopenia, we performed a literature search to identify any autoimmune diseases implicated by the significant HLA types not already included in the autoimmune exclusion criteria. HLA type associations with sarcopenia were reanalyzed after participants with a diagnosis of these further autoimmune conditions had been excluded (Supplementary Table 2); these are the final results presented.

### Sex-Specific Cohort Analysis

HLA types associated with the three definitions of sarcopenia were analyzed with sex-specific cohorts, after the removal of participants with autoimmune conditions. We tested for a statistical interaction between sex and HLA type on sarcopenia, in the models described previously.

### Sensitivity Analyses

Sensitivity analysis was performed on the HLA types associated with sarcopenia across the three phenotypes, for smoking status (UK Biobank data field: 20116) and a combination of height and weight.

### Non-Coding Single Nucleotide Polymorphisms Associated With a Phenotype in Previous Genome-Wide Association Studies

The HLA types assessed so far were based on the protein-coding sequence of the HLA protein expressed. We also investigated non-coding single nucleotide polymorphisms (SNPs) within the HLA region that have previously been associated with traits in the genome-wide association study (GWAS) catalogue; SNPs in the HLA region (4) between GRCh.v38: Chr6:29545629 (start of GABBR1 transcript ENST00000355973.7, minus 10Kb) and GRCh.v38: Chr6:33419924 (end of KIFC1 transcript ENST00000428849.6, plus 10 Kb) were downloaded from the NHGRI-EBI GWAS Catalogue (23). SNPs labeled with one or more of the following contexts were excluded: stop_gained, synonymous_variant, missense_variant, non_coding_transcript_exon_variant, frameshift_variant or inframe_deletion, in order to prioritize variants with a possible regulatory role over variants involved in changes to protein coding. Variants previously reported with an association with any trait (*p*-value < 5*10^–8^; Supplementary Table 3) and had available genotype data in the UK Biobank with a frequency ≥1% in order to account for lower-quality imputation for rarer SNPs, were included in the analysis (*n* = 658). These were tested for their association with sarcopenia using the methods described above for HLA type analysis. We used the Bonferroni adjusted cutoff for 658 tests of *p*-value less than 7.6*10^−5^. Associations between these SNPs and sarcopenia-associated HLA types were also calculated. The GTEx database (24) was used to identify genes with expression affected by the alleles of the genetic variants. A threshold of 3.66*10^–8^ (0.05/4 independent lead SNPs * 341316 GTEx v7 eGenes) was used to select the most significant expression quantitative trait loci (eQTLs).

## Results

We selected 196,099 UK Biobank participants of European descent aged 60–70 with complete phenotype, diagnosis, and genotype data for investigation. Of these, 14,798 participants had at least one diagnosed autoimmune disease (including type 1 diabetes, multiple sclerosis, and rheumatoid arthritis, see “Methods” for details). Participants with a diagnosed autoimmune disease had increased odds of meeting the EWGSOP sarcopenia criteria (odds ratio [OR] 1.83, confidence interval [CI] 1.74–1.92, *p* = 4.0*10^–125^; Table 1). There were specific associations with rheumatoid arthritis (*n* = 2,833): mean grip strength in this group was 22.1 (*SD* 11.3) kg compared to 31.0 (*SD* 10.8) kg (*p* < .001) for all other participants in the European 60–70 age group. Associations were also present for multiple sclerosis, coeliac disease, and psoriasis, but not for type 1 diabetes or ulcerative colitis.

**Table 1.** Selected Autoimmune Conditions and Their Association With the EWGSOP Definition of Sarcopenia (Low Grip Strength and Lean Muscle Mass) in the Overall Sample of 196,099 UK Biobank Participants

Description	Affected, *n*	OR (95% CI)	*p*-Value
Rheumatoid arthritis	4,103	3.09 (2.87–3.34)	6.00*10^–191^
Multiple sclerosis	588	2.14 (1.73–2.64)	1.60*10^–12^
Coeliac disease	1,001	1.82 (1.54–2.17)	6.00*10^–12^
Psoriasis	2,951	1.31 (1.16–1.48)	1.20*10^−05^
Type 1 diabetes	1,565	1.14 (0.95–1.36)	1.70*10^−01^
Ulcerative colitis	2,077	1.13 (0.97–1.32)	1.00*10^−01^
Any autoimmune	14,798	1.83 (1.74–1.92)	4.0*10^–125^

*Note*: CI = confidence interval; EWGSOP = European Working Group on Sarcopenia in Older People; OR = odds ratio. Supplementary Table 10 has full details of additional sarcopenia definitions.

After excluding those with autoimmune diagnoses, the remaining 181,301 participants had a mean age of 64.1 years and 95,340 were female (Table 2). Of 85,961 men included in the analysis, 3,510 were defined as having sarcopenia (4.08%), and of 95,340 women, there were 11,540 defined as sarcopenic (12.10%).

**Table 2.** UK Biobank Participants Characteristics, Excluding Those With Autoimmune Conditions

	*n*	Min–Max	Mean (*SD*)			
Age of study participant (years)	181,301	60–70	64.11 (2.85)			
BMI (kg/m^2^)	181,301	12.81–68.41	27.52 (4.46)			
Grip strength (kg)	181,301	0–73	31.09 (10.71)			
Skeletal muscle mass (kg)	181,301	8.57–46.13	21.96 (6.00)			
			Sarcopenia EWGSOP			
			No		Yes	
	*n*	%	*n*	%	*n*	%
Gender						
Female	95,340	52.59	83,800	87.90	11,540	12.10
Male	85,961	47.41	82,451	95.92	3,510	4.08
Combined	181,301	100.00	166,251	91.70	15,050	8.30
Highest education level attained						
None	48,224	26.60	43,150	89.48	5,074	10.52
Secondary	28,870	15.92	26,096	90.39	2,774	9.61
College level	26,810	14.79	25,006	93.27	1,804	6.73
Professional/university	75,075	41.41	69,882	93.08	5,193	6.92
No data	2,322	1.28	2,117	91.17	205	8.83
Smoking status						
Never	90,242	49.77	82,112	90.99	8,130	9.01
Previous	75,618	41.71	70,031	92.61	5,587	7.39
Current	14,602	8.05	13,356	91.47	1,246	8.53
No data	839	0.46	752	89.63	87	10.37

*Note*: BMI = body mass index; EWGSOP = European Working Group on Sarcopenia in Older People. UK Biobank participants aged 60–70 of European descent with complete grip strength, skeletal mass, genotype (human leukocyte antigen), and autoimmune diagnosis data. Participants with a diagnosis of autoimmune diseases were excluded from analyses.

**Figure 1.**
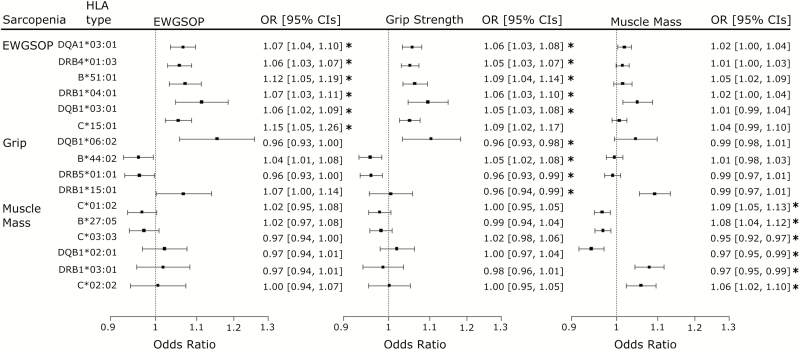
Forest plot of HLA types associated with sarcopenia phenotypes. *Note*: Logistic regression analysis of 100 HLA types associated with sarcopenia (Benjamini–Hochberg correction for multiple testing applied). HLA types passing the false discovery rate cutoff of 0.05 for each phenotype are marked with asterisks (*). CI = confidence interval; EWGSOP = European Working Group on Sarcopenia in Older People, combined sarcopenia definition: low grip strength and muscle mass; HLA = human leukocyte antigen; OR = odds ratio per allele of HLA type.

Of 100 HLA types with allele frequency of more than 1%, 6 were associated with sarcopenia (EWGSOP definition) in logistic regression models adjusted for age, sex, genotyping array type, and population structure (genetic principal components 1–5), after accounting for multiple testing (false discovery rate [FDR] < 5%) (Figure 1). We compared participants with two alleles of each HLA type to those with no alleles; participants homozygous for HLA-DQA1*03:01 have 19.3% (OR 1.19, 95% CI 1.10–1.30, *p* = 2.84*10^–5^) increased likelihood of sarcopenia, compared to those without HLA-DQA1*03:01 (Supplementary Table 4) and HLA-DRB4*01:03 homozygotes had a 15.4% increased likelihood of sarcopenia (OR 1.15, CI 1.08–1.23, *p* = 2.66*10^–5^). When the six HLA types were combined, participants with at least 6 alleles (of a possible 12, each person can have up to 2 alleles of each HLA type) had 23% increased likelihood of sarcopenia (*n* = 5,685 participants of 181,301; OR 1.23, 95% CI 1.12–1.35, *p* = 7.28*10^–6^).

Seven HLA types were associated with the sarcopenia definition based on grip strength alone, and six were associated with the sarcopenia muscle mass definition, with no overlap between the alleles associated with the two phenotypes (Figure 1 and Supplementary Table 5). All significant HLA type associations with the EWGSOP definition of sarcopenia were also present in the low grip strength phenotype, with the exception of HLA-C*15:02 which did not reach significance after correction for multiple testing in the low grip strength phenotype analysis. Conversely, the HLA types associated with low muscle mass (HLA-B*27:05 OR 1.079, 95% CI 1.04–1.12, *p* = 1.64*10^–5^; HLA-C*01:02 OR 1.093, 95% CI 1.05–1.13, *p* = 1.65*10^–6^, and HLA-C*02:02 OR 1.059, 95% CI 1.02–1.10, *p* = 1.46*10^–3^) were distinct from those associated with the EWGSOP definition of sarcopenia.

### Sex-Specific Analysis

We tested for interactions between HLA types and sex on sarcopenia. Two HLA types, HLA-DQA*03:01 (*p* = .032) and HLA-DRB1*04:04 (*p* = .038), were nominally associated with sarcopenia (EWGSOP’s combined definition), indicating that the effect of these HLA types is significantly greater in females, compared to males. The other interactions were not significant, although we cannot rule out lack of statistical power due to the relatively low numbers of men with sarcopenia (Supplementary Table 5).

There were three additional HLA types associated with the EWGSOP’s combined definition of sarcopenia in the female-only analysis (HLA-B*15:01 OR 1.089, 95% CI 1.03–1.15, *p* = 3.27*10^–3^; HLA-DRB1*04:04 OR 1.107, 95% CI 1.03–1.19, *p* = 3.65*10^–3^; and HLA-DQB1*03:02 OR 1.07, 95% CI 1.02–1.12, *p* = 3.2*10^–3^), which were not identified in the analysis including both sexes (FDR-adjusted *p* > .05). In the analysis of males only, there were no significant (FDR-adjusted *p* > .05) associations between HLA types and sarcopenia.

### Sensitivity Analyses

In sensitivity analyses, we investigated the effect of adjusting for height and weight; all the reported risks, increasing associations between HLA types and sarcopenia, remained significant, with nominal changes to effect sizes (Supplementary Table 6). However, the three protective HLA types associated with the sarcopenia grip definition were non-significant after adjusting for height and weight (*p* > .05) in the combined cohort. In the female-only cohort, the protective HLA type DRB1*15:01 was the only HLA type to lose significance. The lean muscle mass-only phenotype maintained the significance of the previously observed HLA types, except in the case of HLA-C*01:02 in the female-only cohort which lost its significant association with sarcopenia.

Adjustment for smoking status had no impact on the associations between HLA types and sarcopenia, for any of the three phenotypes analyzed or across the three cohorts of all participants, female only and male only (Supplementary Table 6).

### Non-Coding SNPs in HLA Region

We analyzed 658 non-coding SNPs (frequency ≥ 1% in order to limit the number of statistical tests undertaken and to avoid misclassification from lower-quality imputation for rarer SNPs) within the HLA region previously implicated in human traits at genome-wide significance (*p* < 5*10^–8^) available in the UK Biobank genotype data. We found 219 SNPs nominally (*p* < .05) associated with the EWGSOP definition of sarcopenia (Supplementary Table 7). Ten SNPs reached the Bonferroni adjusted cutoff of 7.6*10^−5^. Of these, we identified four independent signals (Table 3), after removing those in linkage disequilibrium (*r*^2^ > .4, see Supplementary Table 8). This included rs41268896 and rs2293751 with *p*-values less than 5*10^–8^ (OR 1.079 and 1.069, respectively).

**Table 3.** Non-Coding SNPs Within the HLA Region Associated With EWGSOP Definition of Sarcopenia (*p* ≤ 1.0 × 10^–5^) with gene expression information

RS ID	A1/A0	CHR:POS	Trait	OR	95% CI	*p*-Value	Expressed Gene(s), *p* < 3.66*10^–8^	Tissue Type^a^
rs41268896^b^	A/G	6:32070069	Atopic dermatitis	1.08	1.05–1.11	1.06*10^–8^	ATF6B, CYP21A1P, BAG6, HLA-DQA2	MS, AS, AT, WB
rs2844479	C/A	6:31572956	Height	1.07	1.04–1.09	6.11*10^–7^	BAG6, ATF6B, CSNK2B, LY6G5C	AT, MS, HLV, TH
rs9268645^c^	G/C	6:32408527	Type 1 diabetes	1.06	1.04–1.09	1.50*10^–6^	HLA-DQA2, HLA-DQB2, HLA-DQB1, HLA-DRB6, HLA-DRB9, HLA-DRB1, HLA-DQA1	MS, WB, WB, MS, TH, SE, LN
rs2072633	G/A	6:31919578	Coronary artery disease	1.06	1.03–1.08	3.58*10^–6^	CYP21A1P, ATF6B, HLA-DQA2, PSORS1C1, C2	AS, MS, WB, TH, TE

*Note*: A1 = effect allele; A0 = reference allele; POS = build 37 base pair; trait = the top identified trait from the genome-wide association study catalogue. CI = confidence interval; EWGSOP = European Working Group on Sarcopenia in Older People; HLA = human leukocyte antigen; OR = odds ratio; SNP = single nucleotide polymorphism. GTEx genes for each variant are shown, which reached a threshold of 3.66*10^–8^ for the nominal *p*-value in GTEx (with the most significant tissue type for that expression quantitative trait loci (eQTL) is also shown)—see Supplementary Table 9 for all eQTL associations. Expressed genes in bold are downregulated in GTEx by the effect allele, and all others are upregulated.

^a^Tissue type: AS = adipose subcutaneous; AT = artery tibial; CS = colon sigmoid; EM = esophagus muscularis; HLV = heart left ventricle; LN = lung; MS = muscle skeletal; NT = nerve tibial; SE = sun-exposed lower leg; TE = testis; TH = thyroid; WB = whole blood. ^b^In linkage disequilibrium (LD) with protein QTL for AFT6A (*R*^2^ = .8 with rs8111 in UK Biobank) (25). ^c^Correlated with DQA1*03:01 (*R*^2^ = .42).

We interrogated the GTEx eQTL (expression quantitative trait loci) database of SNP-expression associations to determine the likely genes affected by these four genetic variants (Table 3; see Supplementary Table 9 for full details), using a stringent significance threshold of *p* less than 3.66*10^–8^. This included specific HLA genes, here listed with the tissue in which the strongest eQTL was found (HLA-DQA2—skeletal Muscle, HLA-DQB2—whole blood, HLA-DQB1—whole blood, HLA-DRB6—skeletal muscle, HLA-DRB9—thyroid, HLA-DRB1—skin, sun-exposed lower leg, and HLA-DQA1—lung), in addition to other genes with plausible mechanisms of action in sarcopenia (*ATF6B*—skeletal muscle, *CYP21A1P*—subcutaneous adipose, *BAG6*—tibial artery, *CSNK2B*—left heart ventricle, *LY6G5C*—thyroid, *PSORS1C1*—thyroid, C2—testis). We also searched for protein QTLs in a recent paper by Sun and coworkers (25) and found that rs41268896 is highly correlated (*r*^ 2^ = .8 in UK Biobank study) with rs8111, a pQTL for ATF6A.

We also investigated the combined effect of the four non-coding SNPs; participants with at least four effect alleles (of a possible eight) showed an increased likelihood of sarcopenia of 11% (*n* = 74,820 participants of 181,301; OR 1.11, 95% CI 1.08–1.15, *p* = 1.06*10^–9^).

## Discussion

To the best of our knowledge, this is the first large human population study of HLA effects on sarcopenia in older people. First, we have shown that autoimmune diagnoses are strongly associated with sarcopenia in our sample of 60- to 70-year-old community volunteers, with particularly large effect for rheumatoid arthritis (OR 3.09, 95% CI 2.87–3.34), plus multiple sclerosis, coeliac disease, and psoriasis, but not for type 1 diabetes or ulcerative colitis.

After excluding participants with autoimmune diagnoses, we identified six HLA types associated with sarcopenia in 181,301 UK Biobank participants of European descent aged 60–70. Although these have modest effect sizes (per allele ORs from 1.054 to 1.15), they are common in the study population, ranging in frequency from 3.7% to 24.8%. In combination, participants with more than six of the HLA types had markedly increased likelihood of sarcopenia (OR 1.23, 95% CI 1.12–1.35). We observed markedly different prevalence of sarcopenia in males and females (4% vs 12%); although a recent systematic review found that the average sarcopenia prevalence in males and females was approximately 10% (26), the individual studies varied considerably and it was not unusual to observe substantial sex differences. We performed sex-stratified analysis of HLA types and found that all observed associations were only statistically significant in females, and for some this sex interaction was statistically significant, suggesting that the effect of HLA-DQA1*03:01 and HLA-DRB1*04:04 is substantially greater in females compared to males. Unfortunately, the study lacked the power required for the male-only cohort to observe ORs within the ranges seen in the combined sex analysis.

We also identified four non-coding genetic variants in the HLA region of chromosome six associated with sarcopenia. These are known to affect the expression of multiple genes, including *HLA* genes, in multiple tissues including skeletal muscle. Although more evidence is required for a clear causal link, this suggests that regulation in expression of HLA genes may also be an important driver of sarcopenia, not just the protein sequence (HLA types). When taken in combination, participants with more than four of the effect alleles had an 11% increased likelihood of sarcopenia.

The EWGSOP 2010 definition of sarcopenia (9), which combines low grip strength and low muscle mass, shares many of the associated HLA alleles with those seen in low grip strength definition alone. In contrast, there is little overlap with the low muscle mass definition. Often, grip strength alone is used to identify frail participants in population studies (27) as it is the maintenance of function that appears most important, rather than the loss of muscle mass alone.

HLA types associated with sarcopenia are also associated with conditions linked to joint pain and loss of function, such as rheumatoid arthritis (DRB1*04:01), ankylosing spondylitis (C*15:02), Behcet’s disease (B*51:01, joint swelling and pain prevalence in 45%–60% of cases (28)), and neuropathic pain (DQB1*03:02, although a meta-analysis (29) only showed association to the two-digit allele DQB1*03). Other HLA alleles associated with sarcopenia include DQA1*03:01, which has been linked to pemphigus vulgaris, a painful autoimmune condition which can affect the skin, mouth, and groin with blisters. In addition, a previous study (30) of HLA types and the self-reported fields for rheumatoid arthritis in the UK Biobank has shown an association between DQA1*03:01 and the disease (OR 1.77, CI 1.70–1.85, *p* = 1.5*10^–131^, moderate linkage disequilibrium with HLA-DRB1*04:01 *r*^2^ = .5).

DQB1*03:01 has previously been shown to have an association as a protective allele against primary biliary cholangitis, although our study provides evidence of a novel association with sarcopenia. In this analysis, we excluded participants with a self-reported or hospital diagnosis of autoimmune conditions, including those mentioned here, suggesting that the associations observed here are not due to diagnosed conditions. More work is needed on whether these associations are due to a general pro-inflammatory effect or represent subclinical manifestations of specific autoimmune processes. More work is also needed on whether some patients with sarcopenia have evidence of autoimmune processes and might benefit from targeted treatment.

Some HLA types were protective for sarcopenia: increasing copies of DQB1*06:02, DRB5*01:01, and DRB1*15:01 were all associated with reduced likelihood of sarcopenia, and have previously been implicated in a number of studies in the development of multiple sclerosis (6), although mainly as part of an extended haplotype including all three types.

When we compared participants carrying two alleles of each HLA to those with no alleles, we found far greater effect sizes; for example, the likelihood of being sarcopenic for DQA1*03:01 rises from 6% to 19.3%, suggesting recessive effects. DRB4*01:03 has been reported as appearing with increased frequency in a limited study of Brazilian patients with polyarteritis nodosa (31). Association of DRB4*01:03 with rheumatoid arthritis has mixed evidence in the literature with recent studies showing no association (32), while slightly older and smaller studies in a different population showing a link (33). HLA-DQA1*03:01 has been previously shown to increase the risk of type 1 diabetes (34), when present as part of the DR4 haplotype or in conjunction with DQB*02:01 (35).

The analysis of low grip strength alone reinforced the associations seen in the EWGSOP sarcopenia definition analysis, with only a single additional association for DQA1*01:02. This type was protective against low grip strength and reported as associated with a multiple sclerosis-like condition in transgenic mice (36). HLA alleles were only associated with low muscle mass using the additive model, C*01:02 and B*27:05, both of which have previously been linked to forms of arthritis—psoriatic arthritis (37) and SpA (38). Neither type appeared in the associations for the combined EWGSOP phenotype of sarcopenia, suggesting potential independent effects.

In our analysis of non-coding genetic variation in the HLA region, we identified four genetic variants associated with sarcopenia. These SNPs also affect expression of genes other than HLAs, including *ATF6B*, which encodes a transcription factor in the unfolded protein response pathway during endoplasmic reticulum (ER) stress and there has been speculation that ER stress may impair autophagy and myogenesis activity resulting in sarcopenia (39,40). rs41268896 is strongly correlated with the rs8111 (*r*^2^ = .8), known to affect protein levels of ATF6A (25), which binds as a heterodimer with ATF6B. The rs41268896 A allele increases *ATF6B* expression, while the rs8111 T allele (co-inherited) decreases ATF6A protein levels, suggesting a complex relationship; more work is required to understand this. The *BAG6* protein product is also involved in elimination of misfolded proteins, including class I HLA products (41,42). *CYP21A2* has a role in producing cortisol and aldosterone as part of the hypothalamic–pituitary–adrenal (HPA) axis, and disruption to the HPA has been linked to decline in physical function and aging (43,44).

The strengths of this study include the large number of older participants with consistent sarcopenia measurements and medical record data available for analysis. However, it is a volunteer study and will therefore be healthier than the general population: effects of HLA types on sarcopenia may therefore be underestimated in this study. Future studies should investigate other definitions of sarcopenia, such as those from the FNIH (45) and the revised 2018 version of the EWGSOP definitions (46) which result in differing prevalence (19) due to different cut-points and muscle mass measurements, in older populations. Additionally, autoimmune diagnoses may be under-reported as diagnoses are either self-reported or from hospital in-patient records only; further studies will be required. Future work should clarify the associations between HLA types, circulating inflammatory cytokines, and frailty, ideally in a longitudinal study.

Given the biologically plausible and strong association between autoimmune diseases and sarcopenia, our findings in the autoimmune diagnosis-free group are also plausible. Although all reported associations passed multiple statistical testing correction, our results, especially in men, do require independent replication, as the effects observed may vary between cohorts, although sample size requirements for replication will be challenging.

Functional follow-up work on the HLA types that we have found associated with sarcopenia could give an indication of the underlying mechanisms at work. The mechanisms of effect of the HLA region and specific HLA types in seropositive rheumatoid arthritis (47) and other autoimmune diagnoses have been identified, and it needs to be established whether similar mechanisms explain associations with sarcopenia.

## Conclusions

Autoimmune diagnoses are strongly associated with sarcopenia in 60- to 70-year olds. In older participants without diagnosed autoimmune diseases, we identified six HLA types associated with EWGSOP sarcopenia definition. An additional four unique HLA types were associated with the low grip strength only and six types were associated with the low SMI definition only. Analysis of non-coding variants within the HLA region, which are involved in the expression and regulation of HLA (HLA-DRB1, HLA-DQA2), and the Endoplasmic Reticulum unfolded protein stress response (AFT6B), has shown an association with these variants and sarcopenia. Further studies into the long-term effects of HLA variation are required. More work is needed on whether some patients with sarcopenia have autoimmune processes and might benefit from targeted treatment.

## Funding

This work was generously funded by an award to D.M. by the Medical Research Council (http://dx.doi.org/10.13039/501100000265) MR/M023095/1. D.M. and L.C.P. were supported by the University of Exeter Medical School. (http://dx.doi.org/10.13039/501100000737) Input from C.-L.K. and G.K. was supported by the University of Connecticut Health Center (http://dx.doi.org/10.13039/100007710). L.F. was supported by the Intramural Research Program of the National Institute on Aging, U.S. National Institutes of Health (http://dx.doi.org/10.13039/100000002). This work was supported by an IPA Assignment Agreement with Dr. Luigi Ferrucci at the National Institute on Aging (http://dx.doi.org/10.13039/100000002) (#20170526).

## Conflict of Interest

None reported.

## Supplementary Material

glz042_suppl_Supplementary_Table_1

glz042_suppl_Supplementary_Table_2

glz042_suppl_Supplementary_Table_3

glz042_suppl_Supplementary_Table_4

glz042_suppl_Supplementary_Table_5

glz042_suppl_Supplementary_Table_6

glz042_suppl_Supplementary_Table_7

glz042_suppl_Supplementary_Table_8

glz042_suppl_Supplementary_Table_9

glz042_suppl_Supplementary_Table_10
